# Dating Ammonia-Oxidizing Bacteria with Abundant Eukaryotic Fossils

**DOI:** 10.1093/molbev/msae096

**Published:** 2024-05-22

**Authors:** Tianhua Liao, Sishuo Wang, Hao Zhang, Eva E Stüeken, Haiwei Luo

**Affiliations:** Simon F. S. Li Marine Science Laboratory, School of Life Sciences and State Key Laboratory of Agrobiotechnology, The Chinese University of Hong Kong, Shatin, Hong Kong SAR; Simon F. S. Li Marine Science Laboratory, School of Life Sciences and State Key Laboratory of Agrobiotechnology, The Chinese University of Hong Kong, Shatin, Hong Kong SAR; Simon F. S. Li Marine Science Laboratory, School of Life Sciences and State Key Laboratory of Agrobiotechnology, The Chinese University of Hong Kong, Shatin, Hong Kong SAR; School of Earth and Environmental Sciences and Centre for Exoplanet Science, University of St Andrews, Queen's Terrace, KY16 9TS, UK; Simon F. S. Li Marine Science Laboratory, School of Life Sciences and State Key Laboratory of Agrobiotechnology, The Chinese University of Hong Kong, Shatin, Hong Kong SAR; Earth and Environmental Sciences Programme, The Chinese University of Hong Kong, Shatin, Hong Kong SAR; Institute of Environment, Energy and Sustainability, The Chinese University of Hong Kong, Shatin, Hong Kong SAR

**Keywords:** ammonia-oxidizing bacteria, Great Oxidation Event, molecular clock

## Abstract

Evolution of a complete nitrogen (N) cycle relies on the onset of ammonia oxidation, which aerobically converts ammonia to nitrogen oxides. However, accurate estimation of the antiquity of ammonia-oxidizing bacteria (AOB) remains challenging because AOB-specific fossils are absent and bacterial fossils amenable to calibrate molecular clocks are rare. Leveraging the ancient endosymbiosis of mitochondria and plastid, as well as using state-of-the-art Bayesian sequential dating approach, we obtained a timeline of AOB evolution calibrated largely by eukaryotic fossils. We show that the first AOB evolved in marine Gammaproteobacteria (Gamma-AOB) and emerged between 2.1 and 1.9 billion years ago (Ga), thus postdating the Great Oxidation Event (GOE; 2.4 to 2.32 Ga). To reconcile the sedimentary N isotopic signatures of ammonia oxidation occurring near the GOE, we propose that ammonia oxidation likely occurred at the common ancestor of Gamma-AOB and Gammaproteobacterial methanotrophs, or the actinobacterial/verrucomicrobial methanotrophs which are known to have ammonia oxidation activities. It is also likely that nitrite was transported from the terrestrial habitats where ammonia oxidation by archaea took place. Further, we show that the Gamma-AOB predated the anaerobic ammonia-oxidizing (anammox) bacteria, implying that the emergence of anammox was constrained by the availability of dedicated ammonia oxidizers which produce nitrite to fuel anammox. Our work supports a new hypothesis that N redox cycle involving nitrogen oxides evolved rather late in the ocean.

## Introduction

The nitrogen (N) cycle encompasses a series of transformations between ammonia, dinitrogen (N_2_), and nitrogen oxides, mediated by various biological processes such as N_2_ fixation and nitrification (Yool et al. 2007; Stüeken et al. 2016; Kuypers et al. 2018; Pajares and Ramos 2019; Preena et al. 2021). The evolutionary onset of these processes, especially nitrification (i.e. the oxidation of ammonia to nitrite and nitrate), has important implications for biological activity and other biogeochemical cycles. The production of nitrogen oxides enables biological or abiotic denitrification (i.e. reduction of nitrate and nitrite to N_2_), leading to a depletion of the dissolved N reservoir. It has therefore been hypothesized that the onset of nitrification and denitrification resulted in an N-limited biosphere, which may have impacted microbial evolution (Fennel et al. 2005). Reconstructing the emergence of nitrification in the biosphere is therefore critical for understanding the evolutionary history of life on Earth.

Geochemical techniques, specifically stable N isotopes (δ^15^N) applied to organic-rich sedimentary rocks, provide an indirect tool for constraining biological metabolisms. These data suggest that a dissolved reservoir of nitrate appeared transiently in the Neoarchean surface ocean ca. 2.7 billion years ago (Ga), and the most plausible way to generate dissolved nitrate in significant quantities is by biological nitrification (Godfrey and Falkowski 2009; Koehler et al. 2018). These data are therefore used as indirect evidence for local and/or temporary occurrences of oxygenic photosynthesis (Godfrey and Falkowski 2009; Farquhar et al. 2011). The δ^15^N signal becomes more widespread around the time of the Great Oxidation Event (GOE, 2.4 to 2.32 Ga) (Zerkle et al. 2017; Kipp et al. 2018; Cheng et al. 2019) which marks the permanent oxygenation of the atmosphere and surface environment. The interpretation of the δ^15^N record is consistent with other proxies; however, it is by itself not unambiguous. First, postdepositional processes, such as diagenesis and metamorphism, can alter sedimentary proxies, which creates uncertainty (Robinson et al. 2012; Stüeken et al. 2016; Ossa et al. 2022). Second, and perhaps more importantly, the δ^15^N signal interpreted as evidence of coupled nitrification/denitrification is nonunique and may be mimicked by other metabolisms (e.g. distillation of a dissolved ammonium reservoir by partial assimilation or Fe-based oxidation; Pellerin et al. 2023). Therefore, new techniques are needed to provide independent constraints. Here, we leverage the genomic record of the biosphere, paired with bioinformatic reconstructions of the antiquity of nitrifying bacteria, to determine the age of bacterial nitrification.

The nitrification process is mediated by several microbial functional groups, including the ammonia-oxidizing archaea (AOA) and bacteria (AOB), the nitrite-oxidizing bacteria (Lehtovirta-Morley 2018; Zorz et al. 2018; Park et al. 2020), and complete ammonia-oxidizing bacteria (CAOB or Comammox bacteria) (van Kessel et al. 2015). The bacterial ammonia oxidizers are limited to a few taxa, including members of Betaproteobacteria (Beta-AOB) and Gammaproteobacteria (Gamma-AOB) and the genus *Nitrospira* within the phylum Nitrospirota (Lehtovirta-Morley 2018). Although taxonomically distinct, they use the same enzyme for ammonia oxidation, i.e. ammonia monooxygenase (AMO), which is encoded by the gene cluster *amoCAB* and belongs to the copper membrane monooxygenase (CuMMO) family. The genes encoding the CuMMOs, regardless of the substrate specificity, are referred to as *xmoCAB* (Tavormina et al. 2011; Khadka et al. 2018). Genome-scale analysis focusing on auxiliary genes helps to identify the potential substrate preference of *xmoCAB*-containing bacteria. For example, bacterial ammonia oxidizers utilize the hydroxylamine/hydrazine-ubiquinone-redox-module (HURM) (Klotz and Stein 2008; Simon and Klotz 2013) for energy harvesting. This module requires the presence of two electron carriers, soluble cytochrome *c554* and cytochrome *c_M_552* encoded by the auxiliary genes *cycAB* (Simon and Klotz 2013). Interestingly, pure culture-based studies have reported the ability of methanotrophic bacteria possessing *pmoCAB* (encoding methane monooxygenase) but lacking *cycAB* to oxidize ammonia (O’neill and Wilkinson 1977; Jones and Morita 1983). Because of this substrate promiscuity, the antiquity of ammonia oxidation, if solely estimated based on the *amoCAB* sequences or *amoCAB*-containing microbes, is likely underestimated.

Several studies attempted to date the origin of ammonia oxidizers using molecular clock methods, which assume that the evolutionary rates of protein sequences in different lineages are approximately constant (Zuckerkandl et al. 1962) and employ relaxed molecular clock models to account for rate variations across lineages (dos Reis et al. 2016, 2018). Despite great efforts, the results of previous studies seem often contradictory. Specifically, Ren et al. (2019) estimated that AOA emerged in terrestrial habitats at ∼2.1 Ga and they expanded to oceanic niches at ∼1.0 Ga. Later studies, while agreeing on the ocean-terrestrial habitat transition, suggested a much younger origin of AOA at 1.2 Ga (Yang et al. 2021) or 1.4 Ga (Ngugi et al. 2023). Notably, all the three AOA-timing studies used secondary calibrations (i.e. using previous time estimates, usually in the form of point estimates, as the time priors), which poses the risk that errors in time estimation may easily propagate (dos Reis et al. 2018). In terms of AOB, previous studies (Ward et al. 2021; Gulay et al. 2023) suggested that Beta-AOB appeared earlier than Gamma-AOB and CAOB, therefore postulating a “Beta-AOB early” hypothesis.

Recent molecular dating analyses employed eukaryotic fossils to calibrate bacterial evolution (Wang and Luo 2021, 2023; Liao et al. 2022; Mahendrarajah et al. 2023; Zhang et al. 2023). The inclusion of eukaryotic fossils into bacterial dating is justified by the presence of genes observed to have been shared during ancient endosymbiosis events, notably those involving mitochondria and plastids. These eukaryotic fossils provide many more time boundaries, particularly the maxima, for dating analyses (Clark and Donoghue 2017; Wang and Luo 2023). With the incorporation of two eukaryotic fossils each with a maximum constraint, Liao et al. (2022) estimated the origin of anaerobic ammonia oxidation (anammox) bacteria (AnAOB) at ∼2.3 Ga. However, the authors assumed an unrealistically old age of the crown group of eukaryotes at ∼2.3 Ga (Liao et al. 2022), compared with 1.2 to 1.9 Ga reported by most studies (Parfrey et al. 2011; Betts et al. 2018; Wang and Luo 2021). This leaves the possibility that the age of AnAOB was similarly overestimated.

Here, we leverage extensive eukaryotic fossils through plastid endosymbiosis and mitochondrial endosymbiosis to calibrate the evolution of AOB/CAOB and AnAOB. The Bayesian sequential dating analysis is a two-step molecular clock analysis by first timing the eukaryotes (Fig. 2a) and then imposing their posterior time estimates as calibrations for the second step, which accurately propagates the time information available in the eukaryotic part of the tree to the bacterial nodes. We solve the unrealistically old age problem of the eukaryotes through the Bayesian sequential dating approach. Additionally, we illustrate the impact of using nonvertically transmitted genes on posterior age estimates. Based on a set of predominantly vertically inherited genes, we conclude that AOB predate AnAOB, consistent with the idea that the AOB produce nitrite which serves as the energy source for AnAOB and their electron source for carbon fixation. In contrast to previous studies which reported Beta-AOB as the earliest AOB lineage (Ward et al. 2021; Gulay et al. 2023), our analyses consistently show that Gamma-AOB are the oldest bacterial ammonia oxidizer.

## Results and Discussion

### The Earliest AOB Evolved in Gammaproteobacteria

**Fig. 1.**
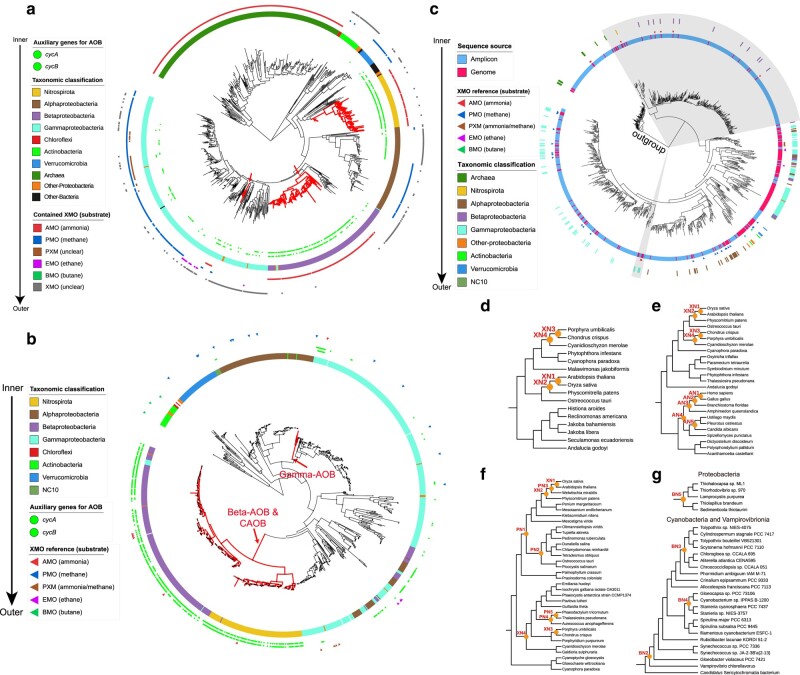
The *xmo* gene phylogeny, the *xmo*-containing genome phylogeny, and the fossil constraints used to calibrate AOB and CAOB evolution. a) The phylogenomic tree of 848 *xmoA*-containing microbes. The phylogenomic tree was constructed using Phylophlan (see supplementary text section S2.3, Supplementary Material online), followed by the outgroup-independent minimum variance (MV) rooting. From inner to outer, the first two rings show the presence of the genes *cycAB*. In the third ring, the color strip represents the major taxonomic lineages derived from the NCBI taxonomy. The remaining six outer rings show the inferred types of XMO according to the concatenated *xmoCAB* gene tree (Fig. 1b). The clades with red branches represent the AOB and CAOB lineages determined based on the presence of *cycAB* and *amoCAB*. b) The concatenated *xmoCAB* gene tree was generated using a total of 964 *xmoCAB* genes, including 922 identified *xmoCAB* operons and 42 reference sequences (Datasets S1.2 & S1.3) at the amino acid level by IQ-Tree (v1.6.12). The gene tree was rooted with the *bmoCAB* lineage following Khadka et al. (2018). The branches inferred as AOB/CAOB were highlighted in red. From the inner to the outer ring, the first color strip indicates the taxonomic affiliation of the concatenated *xmoCAB*. The circles arranged as two rings represent the presence of the auxiliary genes, *cycAB*, which encode the necessary electron carriers for ammonia oxidation and should be present in the ammonia oxidizers. The outmost ring composed of triangles corresponds to the reference sequences encoding XMO for distinct substrates. c) The *xmoA* tree was constructed based on the amino acid sequences of *xmoA* homologs obtained from amplicon sequencing and genome sequencing (see supplementary text section S2.1, Supplementary Material online). The phylogeny was rooted at the *xmoA* sequences from the archaeal lineages, including *Cenarchaeum*, *Candidatus* Nitrosopumilus, and *Candidatus* Nitrosothermus. From the inner to the outer ring, two shaded regions represent the lineages inferred as the *amoCAB* from AOB/CAOB. The first ring represents the type of the sequence, either amplicon or genome sequence. The next ring composed of triangle corresponds to the reference sequences (see supplementary text section S1.1, Supplementary Material online) encoding XMO for distinct substrates. The reference sequences are used to indicate the lineage of XMO with specific substrates. The outermost ring shows the taxonomic affiliation of the tip nodes from genome sequence. Calibration nodes for molecular dating analyses using strategies “Mito24” (d), “Gomez19” (e), “Plastid39” (f), and “Battistuzzi25” (g). The eukaryotic topologies (d, e, and f) were pruned from the topologies provided by the previous studies (see supplementary text section S3.3, Supplementary Material online), and the bacterial topology (g) was pruned from the tree constructed based on the selected 106 genomes (see supplementary text section S2.4, Supplementary Material online). The internal nodes with yellow circles represent the calibration nodes (Datasets S2.1 and S2.2).

We first confirmed the phylogenetic distributions of the bacterial ammonia oxidizers and genes. The phylogenomic tree shows that AOB/CAOB are distributed in three deep taxonomic groups (Fig. 1a). The *xmoCAB* tree (containing *amoCAB* clades) (Fig. 1b) confirmed that *amoCAB* arose twice, once in the last common ancestor (LCA) of the Beta-AOB and CAOB and once in the LCA of Gamma-AOB (Khadka et al. 2018; Lehtovirta-Morley 2018; Soliman and Eldyasti 2018). The two versions of *amoCAB* appear to have evolved independently from their paralogs in the CuMMO family (*xmoCAB* genes) (Fig. 1a). Consistent with a previous study (Osborne and Haritos 2018), we found that the *amoCAB* of Gamma-AOB evolved from the *pmoCAB* of methanotrophs, whereas the *amoCAB* of Beta-AOB and CAOB diversified from the *emoCAB* (ethane monooxygenase) and *pxmABC* (ammonia/methane monooxygenase) of nonmethanotrophs. To investigate the antiquity of bacterial ammonia oxidizers based on their genome sequences, it is essential to confirm that genome sequences of the basal lineages of this functional group are available. We validated this with the *xmoA* gene tree constructed based on *xmoA* sequences associated with genome sequences and derived from amplicon sequencing of the environmental samples, where we show that some of the basal lineages of *amo* genes (shaded regions in Fig. 1c) have representatives derived from genome sequences.

We implemented three strategies (“Plastid39”, “Mito24”, and “Gomez19”; the number informs how many genes were used in each strategy) to compile the molecular data, which was used for the second step of Bayesian sequential dating analysis (dos Reis et al. 2012). They all enable the use of fossil-rich eukaryotic lineages to calibrate fossil-poor bacterial lineages by leveraging the plastid endosymbiosis, the mitochondrial endosymbiosis, and the horizontal gene transfer events that relocated some genes from mitochondria to eukaryotic nuclear chromosomes following the mitochondrial endosymbiosis, respectively. Additionally, we implemented a fourth strategy (“Battistuzzi25”) which exclusively uses the 25 conserved genes in bacteria (*Battistuzzi25* in Dataset S1.5) and the fossil calibrations involving Cyanobacteria and purple sulfur bacteria (PSB). Using either 3.5 Ga or 4.5 Ga as the root maximum (supplementary fig. S4, Supplementary Material online), the bacteria-only strategy “Battistuzzi25”, which does not use eukaryotic fossils and Bayesian sequential dating analysis, estimated the age of the crown Gamma-AOB at 2,019 Ma (95% highest posterior density [HPD]: 1,908 to 2,144 Ma) and 2,310 Ma (95% HPD: 2,160 to 2,465 Ma), respectively. However, all three eukaryote-associated strategies (“Plastid39”, “Mito24”, and “Gomez19”) each gave similar posterior estimates of the crown Gamma-AOB group, with the differences of <10, 20, and 90 Ma, respectively. Therefore, the strategy “Battistuzzi25”, which is heavily affected by the age set for the root maximum, is not further discussed.

**Fig. 2.**
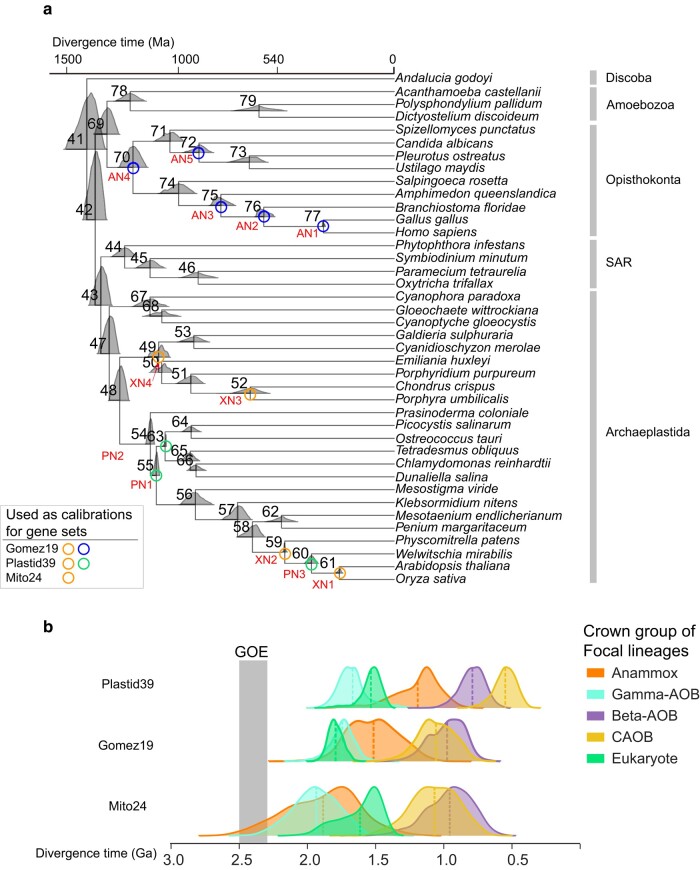
The posterior ages derived from the two-step sequential dating analysis. a) The posterior ages of the eukaryotic nodes derived from the first-step dating analysis. The posterior ages shown in the density plots at each node were estimated based on 320 orthologs and 12 calibrations (see supplementary text section S3.5, Supplementary Material online). The nodes with circles and red calibration node names (XN1 to XN4, PN1 to PN3, and AN1 to AN5) are those with fossil-based calibrations and are those used as calibrations in the second-step dating analysis that includes both eukaryotic and bacterial lineages. The left right panel suggested the used calibrations for each gene set. b) The posterior ages of five focal lineages derived from the second-step dating analysis. Five focal lineages include Gamma-AOB, Beta-AOB, CAOB, Anammox, and eukaryote. The estimation of posterior ages utilizing the 12 best-fit distributions (nodes with circles) that were derived from the first-step dating analysis and employed as priors (see supplementary text section S3.5, Supplementary Material online). Additionally, the bacterial calibration set B1 was utilized in this process (Dataset S2.1). Three gene sets (*Gomez19*, *Mito24*, and *Plastid39*) and corresponding topologies with eukaryotic genomes as mentioned above (Fig. 1) were employed. The dashed line within each density plot represents the mean value of each distribution. The full timetrees are shown in supplementary fig. S3, Supplementary Material online.

Our results consistently support Gamma-AOB as the earliest AOB among the three AOB/CAOB groups, supporting the “Gamma-AOB early” hypothesis (Fig. 2). Specifically, the use of the “Plastid39” strategy gave the posterior ages of the crown groups of Gamma-AOB, Beta-AOB, and CAOB to be 1,667 Ma (95% HPD: 1,429 to 1,868 Ma), 785 Ma (95% HPD: 619 to 979 Ma), and 539 Ma (95% HPD: 397 to 696 Ma), respectively. When the “Mito24” strategy was used, the posterior ages of the three lineages became 1,932 Ma (95% HPD: 1,609 to 2,284 Ma), 961 Ma (95% HPD: 662 to 1,304 Ma), and 1,068 Ma (95% HPD: 737 to 1,401 Ma), respectively. Moreover, using Bayesian sequential approaches, the age of crown group eukaryotes shifted to a younger age (1.6 Ga), widely accepted by most studies (Bengtson et al. 2017; Betts et al. 2018; Javaux 2019), compared to the estimate of 2.3 Ga in Liao et al. (2022) who used the same eukaryote-based calibrations. The reason is likely because the Bayesian sequential molecular clock procedure, as used in the present study, took the advantages of the genome-scale sequence data in eukaryotes thereby better constraining and calibrating the timing of eukaryotes. Further, with the “Gomez19” strategy, we inferred the posterior ages of the three lineages to be 1,740 Ma (95% HPD: 1,571 to 1,944 Ma), 974 Ma (95% HPD: 744 to 1,226 Ma), and 1,057 Ma (95% HPD: 802 to 1,298 Ma), respectively (supplementary fig. S3, Supplementary Material online; Dataset S2.3). The use of the “Plastid39” strategy consistently led to younger estimates of AOB/CAOB compared to the use of “Mito24” and “Gomez19” strategies. This is likely because the “Plastid39” strategy included eukaryotic fossils within Cyanobacteria, whereas the other two strategies subtend eukaryotic lineages to Alphaproteobacteria.

Both Gulay et al.’s (2023) study and our study support the notion that AOB originated following the GOE. However, it is important to note that in contrast to our “Gamma-AOB early” result, previous studies (Ward et al. 2021; Gulay et al. 2023) proposed the “Beta-AOB early” hypothesis. The latter appears to result from the root placement, the taxon sampling, and the lack of calibrations. Gulay et al. (2023) used a phylogenomic tree which places the bacterial root at the total group of the candidate phylum Zixibacteria (a member of Fibrobacteres–Chlorobi–Bacteroidetes group) for dating analysis. The inaccuracy in the root placement could result in the grouping of the Cyanobacteria and Proteobacteria, which is very different from the root placement that separates Terrabacteria (including Cyanobacteria) from Gracilicutes (containing Proteobacteria) by modern phylogenetic methods and models (Hug et al. 2016; Coleman et al. 2021). The reason could be the use of traditional model in their tree building procedure (LG + G); as suggested by recent phylogenomic studies, researchers are highly recommended to use profile mixture models such as C10-C60 in IQ-Tree or CAT in PhyloBayes to account for across-site equilibrium frequency heterogeneity. Another reason for the inconsistent species tree topologies is likely the use of a much larger number of genes in their phylogenetic analysis compared to previous studies (Hug et al. 2016; Coleman et al. 2021) (48 vs. 16 and 29), respectively. This raises the possibility that some of the genes used in Gulay et al.'s study could have undergone horizontal gene transfer, which, if indeed present, could lead to inaccuracy in tree construction. Another study, Ward et al. (2021), did not include a basal lineage of Gamma-AOB represented by the metagenome-assembled genome (GCA_014859375.1); thus, the reported origin of Gamma-AOB at ∼1.0 Ga must be an underestimate. Hence, both studies did not employ the widely used hydrocarbon biomarkers (okenane and other aromatic carotenoids) to constrain the minimum age of PSB (Chromatiaceae) at 1.6 Ga (Brocks et al. 2005). As PSB is a sister lineage of the Gamma-AOB, including the former and its biomarker-based calibration can help constrain the age of the latter. We validated this hypothesis by showing that the lack of the important calibration of PSB resulted in the shift of Gamma-AOB to present over 600 Ma (B1EUK vs. B2EUK or B3EUK vs. B4EUK in Dataset S2.1). Without the time constraint based on this biomarker, Ward et al. (2021) estimated the age of the crown group of Gammaproteobacteria at ∼1.3 Ga and Gulay et al. (2023) estimated the age of the crown group of Chromatiaceae at around ∼1.1 Ga, both of which greatly underestimated the origin time of the PSB and thus probably underestimated the age of AOB.

Resolving the branching order of Beta-AOB and Gamma-AOB is crucial to our reconstruction of the N cycle in early oceans. Since the archaea-driven ammonia oxidation by AOA likely originated in terrestrial systems and did not migrate to the ocean until 1.4 to 1.0 Ga (Ren et al. 2019; Yang et al. 2021; Ngugi et al. 2023), AOA did not contribute to ammonia oxidation in early oceans. Also Beta-AOB members are primarily found in terrestrial environments, and therefore, if the “Beta-AOB early” hypothesis had been true and if the estimates of their origin around 1.9 Ga had been accurate according to Gulay et al. (2023), it would make it difficult to explain the isotopic evidence for a dissolved nitrate reservoir in surface waters throughout most of the Proterozoic (Ader et al. 2016). This enigma is easily resolved by the “Gamma-AOB early” hypothesis, as proposed and validated here, given that Gamma-AOB members are mainly found in marine environments. Therefore, our finding is easier to reconcile with the geochemical record and confirms the interpretation of isotopic data.

### Gamma-AOB Predate AnAOB

**Fig. 3.**
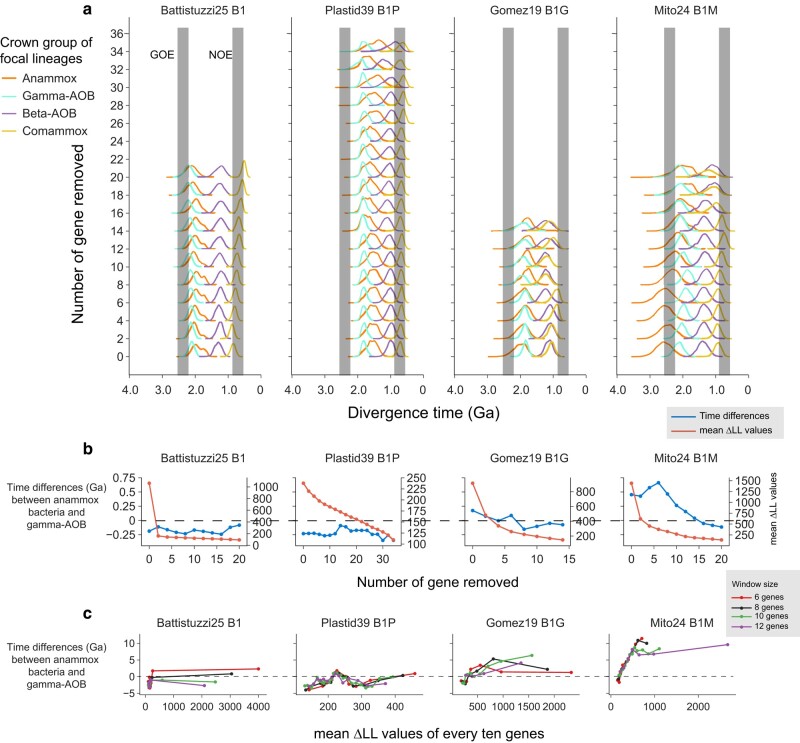
The impact of using phylogenetically incongruent genes on posterior time estimates. For each of the four independent gene sets, the divergence time was estimated using the corresponding calibration set (Dataset S2.1) with MCMCTree (see supplementary text section S3.4, Supplementary Material online). The Bayesian sequential dating approach was not applied for this purpose. The topologies used in each dating analysis (see supplementary text section S3.2, Supplementary Material online) were constructed with bacterial genomes only (“Battistuzzi25”), bacterial genomes and nuclear genomes of eukaryotes (“Gomez19”), bacterial genomes and mitochondrial genomes of eukaryotes (“Mito24”), and bacterial genomes and plastid genomes of eukaryotes (“Plastid39”). a) The change of posterior age estimates of the Gammaproteobacterial AOB (Gamma-AOB), Betaproteobacterial AOB (Beta-AOB), comammox bacteria (CAOB), and anammox bacteria (AnAOB) along with the change of the number of genes by gradually removing two genes that are most topologically incongruent with the species tree (i.e. the largest ΔLL). The time estimates refer to the age of the total group of each lineage. The two vertical gray bars represent the GOE occurring at 2,500 to 2,320 Ma and the Neoproterozoic Oxygenation Event (NOE) at 800 to 550 Ma, respectively. b) The change of the time difference between Gamma-AOB and AnAOB along with the change of the number of genes by gradually removing the two genes with the largest ΔLL. The left *y*-axis shows values calculated by subtracting the mean time estimates of the total group of Gamma-AOB from those of the total group of AnAOB. The right *y*-axis values indicate the mean ΔLL value of the remaining genes following gene removal. c) The scatter plot shows the estimated age difference between Gamma-AOB and AnAOB of analysis using genes selected with the sliding window approach with a window size of *k* genes (*k* = 6, 8, 10, 12) and a step size of one gene. The *x*-axis shows the mean ΔLL of the ten genes selected each time. The *y*-axis shows values calculated by subtracting the mean time estimates of the total group of Gamma-AOB from the mean time estimates of the total group of AnAOB.

Ammonia oxidation, which produces nitrite, the substrate of AnAOB, is considered as the prerequisite for the origin of AnAOB (Canfield et al. 2010; Kuypers et al. 2018; Liao et al. 2022). In a preliminary analysis, we used the full sets of gene families included in the four strategies. We found that the “Plastid39” and the “Battistuzzi25” strategies support this hypothesis, whereas the “Gomez19” and the “Mito24” strategies instead support the opposite trend where AnAOB predate Gamma-AOB. However, when we gradually removed the gene families with large delta log-likelihood (ΔLL) (Shen et al. 2017; Smith et al. 2020), which indicates the removal of genes with low congruence with the species tree (supplementary fig. S1, Supplementary Material online), Gamma-AOB shifted toward the ancient, whereas AnAOB shifted toward the present (Fig. 3a). Once most of the phylogenetically incongruent genes were removed, Gamma-AOB became older than AnAOB (Fig. 3b).

Although this “gene removal” method illustrates the importance of gene verticality on posterior age estimates, it leaves fewer genes for later rounds of molecular dating, and therefore, the factor of gene number is not under control. To solve this problem, we implemented a “sliding window” method to shift the gene sets according to their ΔLL values while keeping the number of genes at 10 (Fig. 3c). The results derived from this approach are consistent with those from the “gene removal” approach, supporting the idea that genes with low ΔLL should be used for molecular clock analysis. In sum, our dating analyses with all the four strategies support the “Gamma-AOB early” hypothesis where Gamma-AOB predate AnAOB (Fig. 2b).

### All Bacterial Ammonia Oxidizers Postdate the GOE But the Origin of Ammonia Oxidation May Be Correlated with the GOE

Geochemical studies using N isotopes proposed a Neoarchean origin of biological nitrification (and denitrification) as early as 2.7 Ga (Garvin et al. 2009; Godfrey and Falkowski 2009; Koehler et al. 2018). The isotopic record further suggests that this metabolism became environmentally more widespread around the GOE at 2.3 Ga (Garvin et al. 2009; Zerkle et al. 2017; Kipp et al. 2018; Cheng et al. 2019). The total and crown group of the Gamma-AOB, however, likely evolved slightly later, at 2.1 Ga (95% HPD: 1,864 to 2,375 Ma; “Mito24” strategy) to 1.9 Ga (95% HPD: 1,766 to 1,970 Ma; “Plastid39” strategy) and from 1.9 Ga (95% HPD: 1,609 to 2,284 Ma; “Mito24” strategy) to 1.7 Ga (95% HPD: 1,429 to 1,868 Ma; “Plastid39” strategy), respectively. At that time, nitrification appears to be well established in the sedimentary isotope record (Kump et al. 2011; Godfrey et al. 2013; Stüeken 2013). The timing may also coincide with a later oxygen-level peak at 1.9 Ga (Large et al. 2022). Hence, Gamma-AOB, which most likely originated in marine ecosystems, given their habitats today (Lehtovirta-Morley 2018), may have contributed to the isotopic records sourced from the marine environment.

There appears to be a mismatch between the oldest proposed geochemical record of nitrification at 2.7 Ga and the origin of Gamma-AOB at 2.1 to 1.7 Ga. One possibility is that the geochemical data prior to the GOE have been misinterpreted due to unrecognized alteration or alternative metabolic reactions. Alternatively, the mismatch of the timing between the expression of aerobic N cycling in the geochemical record and the origin of marine bacterial ammonia oxidizers may reveal that microbes other than the traditional Gamma-AOB contributed to ammonia oxidation prior to the GOE. One possibility is the LCA (node X in supplementary fig. S3, Supplementary Material online) shared by Gamma-MOB and Gamma-AOB, which was proposed to possess a dual *xmoCAB* system (two or multiple copies of *xmoCAB*) capable of performing both methane oxidation and ammonia oxidation (Osborne and Haritos 2018). The crown group of the dual *xmoCAB* system-containing bacteria (node X in supplementary fig. S3, Supplementary Material online) was estimated to arise at 1,867 Ma (95% HPD: 1,766 to 1,970 Ma), 1,905 Ma (95% HPD: 1,773 to 2,054 Ma), and 2,108 Ma (95% HPD: 1,864 to 2,375 Ma) using the strategies “Plastid39”, “Gomez19”, and “Mito24”, respectively. Alphaproteobacterial methanotrophs are unlikely to have contributed to the ammonia oxidation before the GOE, as the crown group of Alphaproteobacteria likely occurred at ∼1.9 Ga which was estimated by leveraging many eukaryotic fossils based on the mitochondrial endosymbiosis (Wang and Luo 2021). However, nonproteobacterial methanotrophs, such as those from the Actinobacteria and Verrucomicrobia phyla, lack time estimations for their antiquity. Despite this, they are known to oxidize ammonia and are considered likely contributors to nitrite accumulation preceding the GOE (Klotz and Stein 2008; Schmitz et al. 2021). Intriguingly, the geochemical evidence of nitrification in the Neoarchean occurs in a time interval that is known for unusually light organic carbon isotope values (δ^13^C_org_) that are generally interpreted as evidence of methanotrophy (Eigenbrode and Freeman 2006). The rise in oxygen concentration has likely led to a decrease in the availability of atmospheric methane, thereby driving methanotrophs, particularly Gammaproteobacterial methanotrophs with particulate methane monooxygenase, to seek alternative substrates such as ammonia. This shift toward ammonia utilization possibly played a role in the expansion of Gamma-AOB.

Another possible ammonia oxidizer in the Neoarchean is AOA. Ren et al. (2019) estimated the origin of the crown and total group of the AOA at 2.1 Ga (95% HPD: 2,060 to 2,285 Ma) and 2.45 Ga (95% HPD: 2,339 to 2,573 Ma). This age bracket allows for the occurrence of AOA a few hundred million years before the GOE at 2.3 Ga. However, these estimates are vulnerable because (i) using secondary calibrations ignores the calibration uncertainty and thereby biases the posterior age estimates (dos Reis et al. 2018; Liao et al. 2022) and (ii) using gene sets without verticality examination results in bias in posterior age estimation, as shown in the present study. Yang et al. (2021) argued that Ren et al. (2019) adopted a strict clock rate model, but this does not seem to us to be correct as Ren et al. (2019) employed an independent rate clock model as evident by the associated source code: https://github.com/luolab-cuhk/thaum-dating-project-2019. Although a more careful analysis is required, it remains well possible that the earliest AOA originated at or before the GOE and contributed to the aerobic N cycle at that time. Because AOA originated on the land and remained restricted to terrestrial environments for over a billion years (Ren et al. 2019) and the sedimentary geochemical records are derived from shallow oceans (Garvin et al. 2009; Pufahl and Hiatt 2012; Zerkle et al. 2017; Kipp et al. 2018), transportation of nitrite from the land to the ocean would be required in this scenario. This interpretation could potentially be reconciled with the rock record, noting that detailed spatial analyses do not exist for these Archean basins, but gradients of nitrate-rich conditions in onshore settings paired with nitrate-depletion offshore have been documented from the mid-Proterozoic (Stüeken 2013; Koehler et al. 2017). Also, transport of nitrate from land to sea by rivers has been proposed in models (Thomazo et al. 2018). Such ammonia-oxidizing settings on land are consistent with some evidence of oxidative weathering starting as far back as 2.95 Ga as suggested by chromium (Cr) isotopic data (Crowe et al. 2013; Frei et al. 2016).

### Change of Genomic Content upon the Origin of Bacterial Ammonia Oxidizers

**Fig. 4.**
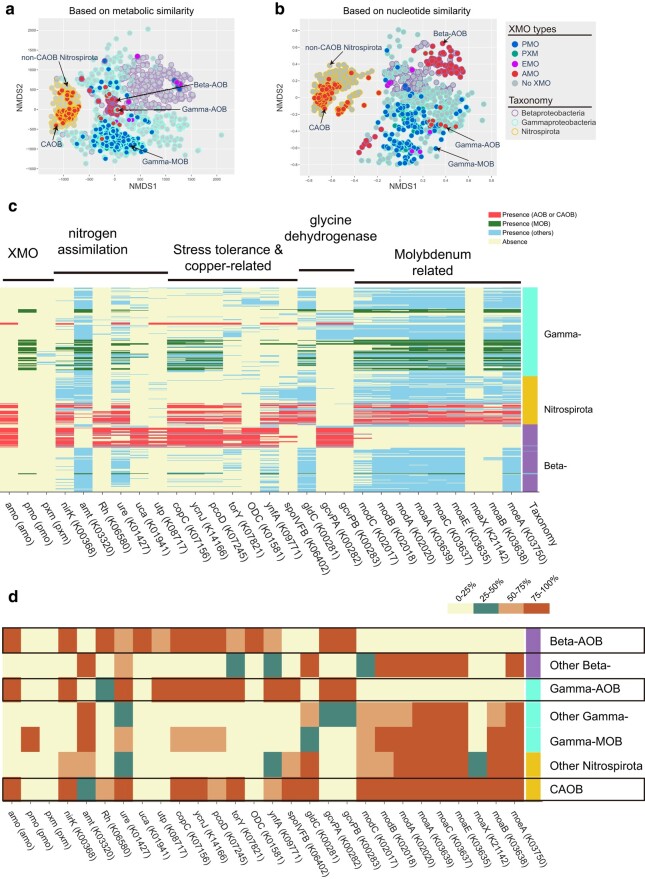
The MDS analyses of 1,119 genomes including *xmoA*-containing genomes and their phylogenetic relatives that do not contain *xmoA*. The MDS analyses were performed based on metabolic similarity according to the presence and absence of KEGG-annotated genes (a) and nucleotide similarity according to the k-mer-based metric (b). Each node represents a genome. The filled color represents the presence and absence of XMO and the type of XMO. The node border color indicates the taxonomic lineages. c) Distributions of genes statistically associated with *amoCAB* among the 1,119 genomes (see supplementary text section S4, Supplementary Material online). The rows and columns represent genomes and KEGG-annotated genes, respectively. To visualize the associations between AMO and other genes, the presence of KEGG-annotated genes in AOB/CAOB, MOB, and other bacteria is highlighted separately. The rightmost column shows the taxonomic affiliation of the genome. d) Summary distribution of *amoCAB*-associated genes. The prevalence of the gene among the genomes in a taxonomic lineage or a focal lineage is categorized into four groups distinguished by four colors. Three focal lineages, namely Betaproteobacterial AOB (Beta-AOB), Gammaproteobacterial AOB (Gamma-AOB), and CAOB, are framed in black boxes. *amo*, ammonia monooxygenase; *pmo*, methane monooxygenase; *pxm*, copper membrane monooxygenase of unknown function; *nirK*, nitrite reductase; *amt*, ammonium transporter; Rh, ammonium transporter; *ure,* urease; *uca*, urea carboxylase; *utp*, urea transporter; *copC*, copper resistance protein C; *ycnJ*, copper transport protein; *torY*, trimethylamine-N-oxide reductase; *ODC*, ornithine decarboxylase; *ynfA*, inner membrane protein; *spoIVFB*, stage IV sporulation protein; *gldC*, homodimeric glycine dehydrogenase; *gcvPAB*, heterotetrameric glycine dehydrogenase; *modABC*, molybdate transport system; *moaABCEX*, molybdenum cofactor biosynthesis; *moeA*, molybdopterin molybdotransferase.

The multidimensional scaling (MDS) plot based on the metabolic dissimilarities grouped the nodes representing Gamma-AOB and Beta-AOB together (Fig. 4a). The normalized metabolic dissimilarity between Gamma-AOB and Beta-AOB is smaller than the normalized nucleotide dissimilarity between them (supplementary fig. S6, Supplementary Material online) (*P* < 0.001; permutation test), suggesting convergent evolution of metabolic changes from non-AOB to AOB in the two independent origins (Beta-AOB and Gamma-AOB). However, the nodes representing CAOB are closer to the non-CAOB Nitrospirota than to the proteobacterial AOB in both MDS plots (Fig. 4a & 4b). Since CAOB nodes are evolutionarily distant from AOB nodes, but *amoCAB* of CAOB and Beta-AOB form a monophyletic lineage (Fig. 1b), a reasonable evolutionary scenario is that CAOB acquired its *amoCAB* horizontally from Beta-AOB (Palomo et al. 2018) according to the tanglegram (supplementary fig. S7, Supplementary Material online) while keeping most of its genomic regions similar to their non-CAOB Nitrospirota counterparts (Palomo et al. 2022).

We implemented three association tests to investigate the genomic changes that occurred between non-AOB and AOB as well as between non-CAOB Nitrospirota and CAOB (see supplementary text section S4.2, Supplementary Material online). With regard to N-related metabolisms, we found the presence of the *nirK* in both Gamma-AOB and Beta-AOB, but not in non-AOB close relatives. In contrast, nearly all Nitrospirota (non-CAOB and CAOB) contain *nirK*. The *nirK* gene, which encodes nitrite reductase that is commonly used for reducing nitrite to nitric oxide in denitrification, here is associated with the *amo* gene cluster (Fig. 4c). During ammonia oxidation, the *nirK* nitrite reductase is used to oxidize nitric oxide to nitrite (Wijma et al. 2004; Caranto and Lancaster 2018; Lancaster et al. 2018) and promotes ammonia oxidation at low oxygen levels (5%) (Kozlowski et al. 2014). Therefore, if *nirK* evolved no later than the origin of bacterial ammonia oxidizers, it would play an important role in facilitating the emergence of bacterial ammonia oxidizers in early ocean where oxygen levels were very low.

Regarding ammonia transportation and utilization, the *amt* gene for high-affinity ammonia transportation (Khademi et al. 2004) is replaced by the *rh* gene for low-affinity transportation in AOB (Kitano and Saitou 2000; Lupo et al. 2007). Specifically, the majority of non-AOB contain *amt*, while the majority of AOB possess the *rh* gene (for low-affinity transportation) (Fig. 4c). Note that this replacement is also found between non-CAOB and CAOB, which reinforces its importance for bacterial ammonia oxidizers. The primary use of *rh* rather than *amt* in AOB and CAOB (Stein et al. 2007) is likely because the former facilitates bidirectional translocation of ammonia across the cell membrane, which might help adapt to both oligotrophic (importer) and eutrophic (exporter) environments. Furthermore, the *rh* transporter works best in neutral to alkaline conditions (Weidinger et al. 2007; Sogaard et al. 2009) where ammonia, the substrate of aerobic ammonia oxidation, is more available than ammonium (Ullmann et al. 2012).

Both AOB and CAOB can perform ammonia oxidation with urea as the substrate (Koper et al. 2004; Zorz et al. 2018) (Fig. 4c) because they are equipped with metabolic pathways that yield ammonia from urea. This could be either a one-step pathway that uses urease hydrolase encoded by the gene *ure* (Callahan et al. 2005) or a two-step pathway that comprises urea carboxylase and allophanate hydrolase encoded by *uca* and *atzF*, respectively (Kanamori et al. 2004). The *ure* is found in most bacteria, and the gene *uca* is uniquely found in Beta-AOB. Note that *atzF* is present in almost all Proteobacteria (Aukema et al. 2020) including the genomes analyzed here (not shown in Fig. 4). Additionally, the gene encoding urea transporter, *utp*, is found in Gamma-AOB and Beta-AOB and missing in CAOB.

Apart from ammonia, copper serves as a main co-factor in AMO and may play an important role in facilitating the origin of bacterial ammonia oxidizers. Marine copper levels sourced from hydrothermal vents and oxidative weathering may have increased during the GOE (Stüeken 2020). The presence of copper in a range of 0.05 to 5 μg/L could significantly increase the rate of ammonia oxidation (Wagner et al. 2016), but a higher concentration of copper could inhibit the ammonia oxidation (Cela and Sumner 2002). This emphasizes the significance of enzymes in promoting tolerance and preserving equilibrium, such as resistance proteins and transporters. Three copper-related genes show strong correlations including *copC* (copper resistance protein C), *pcoD* (copper resistance protein D), and *ycnJ* (copper transport protein) (Chillappagari et al. 2009). They were found in AOB and CAOB, but not in their non-AOB relatives (Fig. 4). Note that copper also plays an important role in *pmoCAB*, and indeed, these copper-related genes were also found in around 50% of MOB (Fig. 4d). Likewise, molybdenum is the cofactor of nitrite oxidoreductase (NXR) and nitrate reductase (NAR) (Schwarz and Mendel 2006; Miralles-Robledillo et al. 2019), which are involved in the reversible transformation between nitrite and nitrate carried out by NAR and/or NXR. Intriguingly, the genes for molybdenum cofactor (molybdopterin) biosynthesis (*moaABCXE*, *moeA*) and molybdate transport (*modABC*) are present in CAOB but missing from Gamma-AOB and Beta-AOB (Fig. 4). The alternative gene (*moaF*) for molybdopterin biosynthesis is absent from the sampled genomes. The presence of molybdopterin synthesis genes in CAOB probably helps the former to synthesize the cofactor for NXR and help complete the ammonia oxidation from ammonia to nitrate. Marine molybdenum levels increased across the GOE and again in the Neoproterozoic (ca. 0.6 Ga; Scott et al. 2008), which may be related to the late origin of CAOB and the colonization of CAOB in diverse ecosystems (Sun et al. 2021; Palomo et al. 2022).

### Concluding Remarks

Gamma-AOB are known to have originated in marine environments (Lehtovirta-Morley 2018). Here, we added that they are also the earliest bacterial ammonia oxidizers among the known AOB/CAOB lineages. This finding aligns with the fact that most N isotopic data indicative of nitrification occur in marine sedimentary strata (Zerkle et al. 2017; Kipp et al. 2018). The origin of Gamma-AOB coincides with a time when N isotopes suggest widespread presence of nitrification. Yet, members of the Gamma-AOB crown and total groups, which originated after the GOE (ca. 2.3 Ga), were unlikely to be direct contributors to the earliest isotopic signatures of nitrification, which date as far back as 2.7 Ga. One possibility is that the first ammonia oxidizer was the common ancestor of Gamma-AOB and Gamma-MOB (Osborne and Haritos 2018), given that the latter also has ammonia oxidation activities. Furthermore, other types of methanotrophs, such as those from the Verrucomicrobia and Actinobacteria phyla, have been postulated to possess the capacity for ammonia oxidation (Stein and Klotz 2011; Schmitz et al. 2021). Although the antiquity of these specific methanotrophs was not explored in this study, the specialized extreme environmental niches inhabited by Verrucomicrobia suggest that they could have arisen prior to the GOE. Intriguingly, the carbon isotope record at that time indicates widespread methanotrophy (Eigenbrode and Freeman 2006), opening up the possibility that the origin of methanotrophs went hand in hand with the origin of ammonia oxidation. Another possible contributor is some early AOA. Although AOA originated in terrestrial environments and remained there until well into the mid-Proterozoic (Ren et al. 2019), it is possible that nitrate was generated on land and transported to marine habitats, consistent with evidence of oxidative weathering in the Archean (Crowe et al. 2013; Frei et al. 2016). Moreover, although AOA dominate the ammonia-oxidizing communities in the modern ocean (Francis et al. 2005), Gamma-AOB was likely the only ammonia oxidizer in the earlier ocean before AOA expanded its habitat to the oceans.

Of the processes that constitute the N cycle, only nitrification uses oxygen as a substrate (Kuypers et al. 2018). Yet, anaerobic N metabolisms heavily rely on ammonia oxidation to provide nitrite as a substrate to fuel the anaerobic processes. For example, aerobic ammonia oxidation and anammox are usually coupled in modern ecosystems (Jenni et al. 2014; Lackner et al. 2014; Liu et al. 2020), which implies their evolutionary order, whereby the former process facilitates the production of nitrite as a substrate for the latter. The conclusion drawn from our analyses supports the idea that Gamma-AOB emerged earlier than AnAOB, which reinforces the hypothesis of a stepwise emergence of these pathways. The ammonia oxidation carried out by methanotrophs prior to the GOE was likely limited to oxygen oases where methane levels may have been low, because the presence of high methane concentrations may suppress this ammonia oxidation (Bodelier and Laanbroek 2004). The decline of methane after the GOE may thus explain why ammonia oxidation expanded and radiated into other phyla, including Gamma-AOB, and later enable the appearance of AnAOB. Likewise, nitrite oxidation, dissimilatory nitrate reduction to ammonium, and denitrification ultimately depend on nitrite produced by aerobic ammonia oxidation. The Proterozoic appearance of Gamma-AOB may therefore explain the expansion of these metabolisms across the tree of life around the same time (Parsons et al. 2021). Our results indicate that nitrate became a more widely available substrate in the Proterozoic, possibly with important consequences for nitrate-assimilating eukaryotes (Anbar and Knoll 2002), the production of N_2_O greenhouse gas (Buick 2007), and the loss of fixed N from the global ocean (Fennel et al. 2008).

## Materials and Methods

The AMO homologs, *xmoCAB* genes, were retrieved by BLASTP using reference protein sequences against the nr database (last updated July 2021). The retrieved 82,848 *xmoA* homologs were clustered by CD-HIT (v4.8.1) (Fu et al. 2012) using 0.9 as the sequence identity threshold, then aligned using MAFFT (v7.222) (Katoh and Standley 2013), and trimmed by ClipKit (v1.1.5) (Steenwyk et al. 2020). The protein mixture model (LG + C60) and ultrafast bootstrap test implemented in the IQ-Tree (v1.6.2) (Nguyen et al. 2015) were used to create the gene tree for *xmoA* (Fig. 1c). According to the source of retrieved *xmoA* homologs, we compiled a genome set which include both *xmoCAB*-containing genomes and their closes relatives (7,683 in total; Dataset S1.1) (see supplementary text section S1.2, Supplementary Material online) via Entrez (Kans 2020) and a custom python script (see Data and Code availability). Moreover, the auxiliary genes *hao*, *cycAB*, and *nxrAB* were annotated by using BLASTP (E-value < 1e−50) based on the reference sequences. The phylogenomic tree (Fig. 1b) comprising 848 *xmoA*-containing genomes (Dataset S1.3) was generated by PhyloPhlAn (v3.0) (Segata et al. 2013) using “high diversity” mode. We then identified the genomes containing at least two genes of the *xmoCAB* gene cluster using a custom python script and concatenated the *xmoCAB* alignments for phylogeny reconstruction using IQ-tree under the auto-determined substitution model (LG + C50 + G) with 1,000 ultrafast bootstrap replicates. The LCAs of the three groups of ammonia oxidizers (i.e. Gamma-AOB, Beta-AOB, and CAOB) are recognized based on the presence and absence of the *cycAB* (Fig. 1b) and *xmoCAB* genes.

Due to the large computational cost of Bayesian-based molecular dating analysis, we performed a two-step taxon sampling strategy (see supplementary text section S2.4, Supplementary Material online) to select a total of 106 bacterial genomes and 75 eukaryotic genomes for our dating analysis. The inclusion of archaeal genomes for AOA dating would reduce the number of genes used in the molecular dating analysis. Moreover, timing the origin of archaea and bacteria together would incur several controversial issues, such as root placement and substitution model selection. Therefore, the archaeal genomes were not used in the dating analysis. For Bayesian dating analysis, four gene sets were compiled including, (i) “Battistuzzi25”: 25 universally conserved genes among bacteria (Battistuzzi and Hedges 2009), (ii) “Mito24”: 24 conserved genes encoded by mitochondrial genomes (Wang and Luo 2021), (iii) “Gomez19”: 19 mitochondria-originated genes that have likely been transferred to the eukaryotic nuclear genome originally identified by (Munoz-Gomez et al. 2022) and that are conserved across the bacterial tree of life (Wang and Luo 2023), and (iv) “Plastid39”: 39 genes that are conserved among eukaryotic plastids, cyanobacteria, and our genome sets (Ponce-Toledo et al. 2017). Note that 39 genes were selected out of 79 genes that are conserved among eukaryotic plastids and cyanobacteria based on the distributions of the genes in our genome sets. We searched these marker genes in our genome sets by using HMMER (v3.2.1) (Mistry et al. 2013) or BLASTP 2.9.0 (Johnson et al. 2008) with the *E*-value threshold at 1e−20 (see supplementary text section S3.1, Supplementary Material online). The presence and absence of these marker genes are shown in Dataset S1.8.

The molecular dating analysis was performed by using the program MCMCTree in the PAML package (4.9j) (Yang 2007). The species tree topology used in molecular dating analysis was first reconstructed with 106 bacterial genomes using the LG + C60 + F + G model with IQ-Tree and then expanded by incorporating representative species that used in previous studies (see supplementary text section S3.3, Supplementary Material online) using ETE (Huerta-Cepas et al. 2016) (Fig. 1d to g). To select the best-fit clock model, we conducted an approximate likelihood method (dos Reis and Yang 2011) by using MCMCTree and calculated the Bayes factor of the independent rate model and the auto-correlated rate model. In order to evaluate the impact of gene family verticality on posterior time estimates, we employed the “gene removal” and the “sliding window” method to compile a series of subset of molecular data based on the ΔLL values of the gene families in each strategy (see supplementary text section S3.4, Supplementary Material online). The gene removal approach step-wisely removes genes with larger ΔLL, while the sliding window approach uses every ten genes following the sorting of all genes according to their ΔLL values. Then, we employed Bayesian sequential strategy (dos Reis et al. 2012) to refine the bacterial timeline estimation using the gene sets “Plastid39”, “Gomez19”, and “Mito24” (see supplementary text section S3.5, Supplementary Material online). We performed the first-step dating analysis using 40 eukaryotic nuclear genomes (Dataset S1.7), 320 orthologs (Strassert et al. 2021), and 14 calibrations (XN1 to XN4, PN1 to PN3, and AN1 to AN5 in Dataset S2.1). The estimated posterior ages were subsequently used as the time priors for the second-step dating analysis for “Plastid39”, “Gomez19”, and “Mito24”, respectively. The convergence of the molecular dating analysis was evaluated by comparing the posterior dates of two independent runs.

To identify the metabolic differences between AOB and non-AOB and between CAOB and non-CAOB, we sampled 1,149 genomes from GenBank (supplementary text section S4.1, Supplementary Material online). We then assessed the dissimilarities between genomes using non-metric MDS algorithm, which quantifies the metabolic dissimilarity and the nucleotide dissimilarity by using the pairwise Manhattan distance matrix and the pairwise mash distance matrix, respectively (Ondov et al. 2016). The protein-coding genes of the sampled genomes were annotated by using Kyoto Encyclopedia of Genes and Genomes (KEGG) (Aramaki et al. 2020) (see supplementary text section S4.1, Supplementary Material online) and then subjected to Fisher’s exact test, Pearson’s correlation test, and the phylogenetic signal test (phylosig) (Blomberg et al. 2003; Revell 2012) to identify their associations with *amoCAB*. For each gene, the tests were made between (i) AOB, CAOB, and others, (ii) AOB and non-AOB Proteobacteria, (iii) Gamma-AOB and non-AOB Gammaproteobacteria, (iv) Beta-AOB and non-AOB Betaproteobacteria, and (v) CAOB and non-CAOB Nitrospirota. The resulting *P*-values were corrected using the Benjamini–Hochberg false discovery rate procedure. Finally, genes with corrected *P*-values <0.05 in five groups of comparisons are manually examined according to the correlation coefficient and the phylogenetic signal derived from the R package “phylosig” (Revell 2012).

## Supplementary Material

msae096_Supplementary_Data

## Data Availability

The input and output files of our dating analysis and the custom scripts are deposited in the online repository https://github.com/luolab-cuhk/AOB-origin.
